# The effects of music therapy in patients undergoing septorhinoplasty surgery under general anesthesia^☆^

**DOI:** 10.1016/j.bjorl.2019.01.008

**Published:** 2019-03-05

**Authors:** Erhan Gökçek, Ayhan Kaydu

**Affiliations:** Diyarbakır Selahaddin Eyyübi State Hospital, Department of Anesthesiology and Reanimation, Diyarbakır, Turkey

**Keywords:** Music therapy, General anesthesia, Pain, Postoperative recovery, Terapia musical, Anestesia geral, Dor, Recuperação pós-operatória

## Abstract

**Introduction:**

Music has been used for several years as a relaxation method to reduce stress and anxiety. It is a painless, safe, inexpensive and practical nonpharmacologic therapeutic modality, widely used all over the world.

**Objectives:**

We aimed to evaluate the effect of music therapy on intraoperative awareness, patient satisfaction, awakening pain and waking quality in patients undergoing elective septorhinoplasty under general anesthesia.

**Methods:**

This randomized, controlled, prospective study was conducted with 120 patients undergoing septorhinoplasty within a 2 months period. The patients were randomly selected and divided into two groups: group music (music during surgery) and control group (without music during surgery). All patients underwent standard general anesthesia. Patients aged 18–70 years who would undergo a planned surgery under general anesthesia were included. Patients who had emergency surgery, hearing or cognitive impairment, were excluded from the study.

**Results:**

A total of 120 patients were enrolled, and separated into two groups. There were no statistically significant differences between the groups in terms of demographic characteristics, anesthesia and surgery durations (*p *> 0.05). In the music group, sedation agitation scores were lower than those in the control group at the postoperative period (3.76 ± 1.64 vs. 5.11 ± 2.13; *p *< 0.001). In addition; in patients of the music group, the pain level (2.73 ± 1.28 vs. 3.61 ± 1.40) was lower (*p *< 0.001), requiring less analgesic drugs intake.

**Conclusion:**

Music therapy, which is a nonpharmacologic intervention, is an effective method, without side effects, leading to positive effects in the awakening, hemodynamic parameters and analgesic requirements in the postoperative period. It is also effective in reducing the anxiety and intraoperative awareness episodes of surgical patients.

## Resumo

**Introdução:**

A música tem sido usada há vários anos como um método de relaxamento para reduzir o estresse e a ansiedade. É um método de tratamento não farmacológico, seguro, barato e prático, amplamente usado em todo o mundo.

**Objetivo:**

Avaliar o efeito da musicoterapia no despertar intraoperatório, na satisfação do paciente, na dor ao despertar e na qualidade de vigília em pacientes submetidos à rinosseptoplastia eletiva sob anestesia geral.

**Método:**

Estudo prospectivo, randomizado e controlado feito com 120 pacientes submetidos a rinosseptoplastia em 2 meses. Os pacientes foram selecionados aleatoriamente e divididos em dois grupos: musicoterapia (música durante a cirurgia) e controle (sem música durante a cirurgia). Todos os pacientes foram submetidos a anestesia geral padrão. Pacientes entre 18 e 70 anos que seriam submetidos a cirurgia planejada sob anestesia geral foram incluídos. Pacientes submetidos a cirurgia de emergência, apresentavam deficiência auditiva ou cognitiva foram excluídos do estudo.

**Resultados:**

Foram inclu¡dos no estudo 120 pacientes, divididos nos dois grupos. Não houve diferenças estatisticamente significantes entre os grupos em relação às características demográficas, anestesia e duração da cirurgia (p > 0,05). No grupo musicoterapia, os escores de agitação da sedação foram menores do que no grupo controle no período pós-operatório (3,76 ± 1,64 vs. 5,11 ± 2,13; *p* < 0,001). Além disso, nos pacientes do grupo musicoterapia, o nível de dor (2,73 ± 1,28 vs. 3,61 ± 1,40) foi menor (*p* < 0,001) e a necessidade de analgésicos foi menor no pós-operatório.

**Conclusão:**

A musicoterapia, uma intervenção não farmacológica, é um método eficaz, sem efeitos colaterais, que leva a efeitos positivos no despertar, nos parâmetros hemodinâmicos e nas necessidades analgésicas no pós-operatório, além de reduzir a ansiedade por estresse, a dor e a chance de despertar durante a cirurgia.

## Introduction

The rapid development of anesthesia techniques in the recent years, has gradually expanded the working areas of anesthesiologists outside the operating room, and the increase of the number of daily operations, have lead to an increase of the patients expectations regarding safety and comfort. Anesthesiologists are responsible for ensuring the safety and comfort of the patient before, during and after the operation, especially inside the operation room. The increasing responsibilities, the expectations of patients and their relatives force us to update our knowledge in anesthesia practice and to develop new methods.^1^, ^2^

Almost all of the patients to be operated present with anxiety, begins at least two days before the surgery. The anxiety gradually increases in the operation room accompanied by feelings of fear, doubt and desperation.^3^ Increases in respiratory rate, heart rate, blood pressure, plasma adrenaline and noradrenalin levels are some physiological responses to pre-surgical stress and anxiety. In addition to operations; the removal of anxiety observed in almost all the patients during the diagnostic or invasive procedures with a proper premedication has become a routine practice.^4^, ^5^ The aim of the sedation is to bring the patient who is confronted with anxiety and painful attempts to a position where they can safely and calmly undergo the anesthesia and surgical procedures. However, this is difficult to obtain because of the variability of the sedation level, expectations of the patients, difference in the intraoperative conditions, and the different pharmacokinetic and pharmacodynamic properties of the used agents.^6^

Currently, pharmacologic treatment options for the perioperative period anxiety and pain, and complementary medical interventions such as hypnosis, acupuncture and music therapy, are becoming increasingly more popular, even if the results are not yet completely known yet. Music has been used for several years as a relaxation method to reduce stress and anxiety. It is a painless, safe, inexpensive and practicable nonpharmacologic treatment, widely used all over the world.^7^, ^8^, ^9^

Many studies have employed music therapy and other therapeutic suggestion methods, and it has been observed that the patients’ surgical anxiety has decreased and a sedative effect has been observed in the perioperative period. In addition to the anxiolytic and sedative effects, this therapy has shortened the duration of postoperative recovery and has reduced the need for analgesic drugs. Also, it has been reported that listening to music reduces the need for sedative drugs and improves satisfaction in patients undergoing regional anesthesia.^10^, ^11^ In a few studies conducted in patients under general anesthesia, it has been concluded that music and intraoperative therapeutic suggestions have positive effects on postoperative recovery and analgesic consumption.^11^, ^12^, ^13^

We aimed to determine the sedative effects of music by preventing the anxiety caused by the noise in the operating room in patients who underwent septorhinoplasty under general anesthesia and to investigate the effects of music on perioperative respiratory and hemodynamic parameters, analgesic consumption and occurrence of intraoperative awareness.

## Material and methods

### Patients

**Figure 1:**
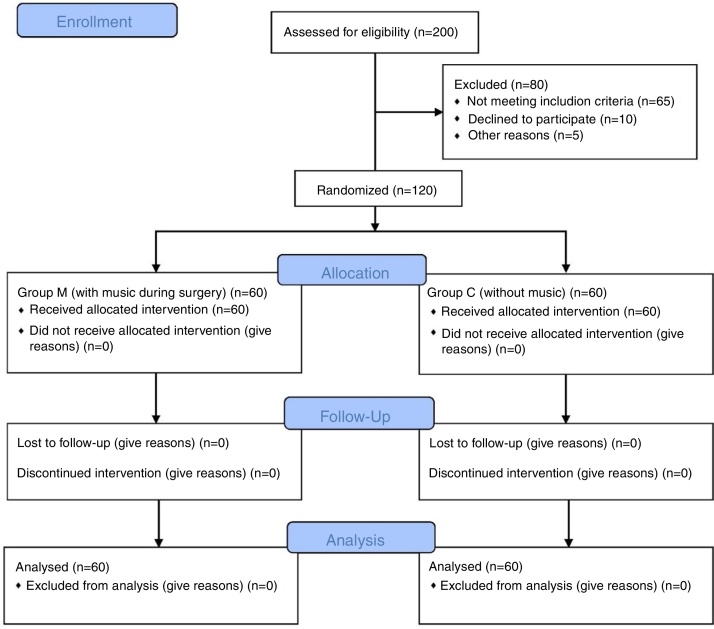
Flow chart.

This randomized, double-blind, and prospectively research was conducted in a single urban state hospital. The approval for the research was granted by the Institutional Ethics Committee (decision no. 2017/72, Gazi Yasargil Training and Research Hospital Ethics Committee). Written and spoken informed consent was obtained from all patients. A total of 120 patients between the ages of 18 and 70 years undergoing septorhinoplasty under general anesthesia were included in this study. Their demographic characteristics, ASA classification, age, height (cm) and weights (kg) were recorded. Patients with hearing problems, those unable to cooperate (due to dementia, mental retardation, etc.), those with drug or alcohol abuse history, and those who did not want to participate in the study were excluded (Fig. 1).

### Preoperative procedure

**Figure 2:**
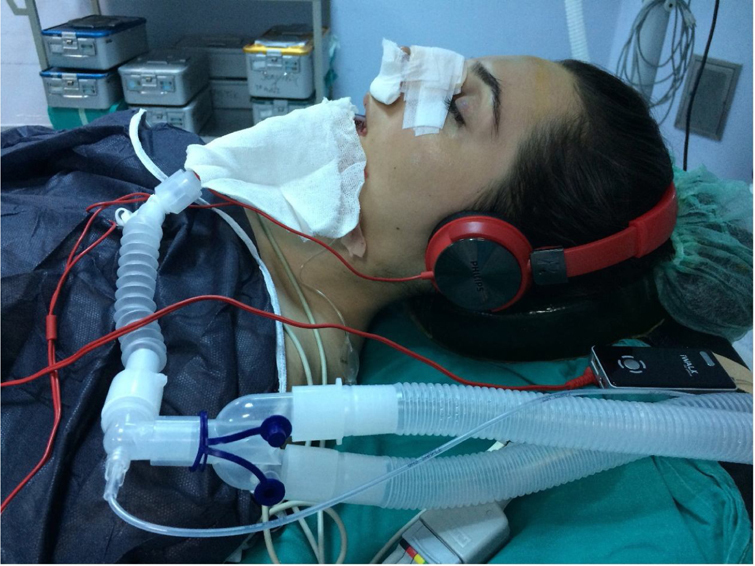
The application of music therapy under general anesthesia.

The patients were randomized into two groups: music group (group M, *n* = 60) and control group (group C, *n* = 60). Only patients in the music group put on headphones (Philips, SHP1900) enclosing the ears, preventing patients from hearing the voices and noises inside the operation room. The intensity of the music sound was set at a level (65 decibels with a standard sound level meter) in which patients would feel comfortable when asked. During the whole operation, all the patients in the music group listened to relaxing indigenous and foreign music (pop, arebesk, jazz, alaturka, classical, ethnic, MMP-3078) by a mp3 (Mpeg-1 Audio Layer 3) device according to their preferences, until the anesthetic gases are initiated. Classical music was chosen by the anesthesiologist for the patients who did not show any specific preference (Fig. 2).

### Anesthesia management

Thirty minutes before surgery, all patients were premedicated with 0.03 mg/kg intramuscular midazolam (Dormicum®, Roche). During the anesthesia induction, 2.5 mg/kg IV propofol (Pofol®), 1 μg/kg fentanyl (Fentanyl®, Janssen) bolus IV, and 1 mg/kg IV arithmetic (Aritmal®, Adeka) were applied. Muscle relaxation was achieved with 0.6 mg/kg rocuronium (Esmeron®, 10 m/mL, Organon). After endotracheal intubation, 2% sevoflurane (Sevorane®, Abbot) was injected with 40% oxygen rate in anesthesia maintenance. In addition to inhalation anesthesia, IV infusion of remifentanil (Ultiva, Glaxo Welcome) was applied at 0.05–10 μg/kg/min. The remifentanil dose was increased or decreased when an increase or decrease of more than 20% of the baseline systolic arterial pressure was observed. An additional muscle relaxant was administered depending on the duration of the operation and follow-up of the neuromuscular blockade. When the heart rate dropped below 50 beats/min, 0.5 mg atropine was injected; when mean arterial pressure (MAP) dropped below 60 mg, 10 mg of ephedrine was injected.

Patient's routine electrocardiography (ECG), noninvasive blood pressure and peripheral oxygen saturation (SpO2) were continuously monitored. Measurements of heart rate, systolic arterial pressure (SAP), diastolic arterial pressure (DAP), and mean arterial pressure (MAP), peripheral oxygen saturation, and baseline values were recorded. Heart rate, SAP, DAP, MAP, SpO2, and minimum alveolar concentration (MAC) values were recorded on induction, intubation, every 5 min of anesthesia, every 15 min and immediately after extubation. All measurements were accomplished with Datex-Ohmeda anesthesia equipment (AS/3, Datex®, Helsinki, Finland). The period from the beginning of the anesthesia induction until the moment the patient was taken to the recovering room, was defined as the duration of the anesthesia; and the period of time from the surgical incision to the skin closure was defined as the duration of the surgery.

### Postoperative procedure

After extubation, patients were taken to the postanesthetic care unit (PACU) for 30 min, to be evaluated. ECG, noninvasive blood pressure and SpO2 monitors were analyzed.

### Data collection

Sedation scores were recorded at 0, 5, 15, 30 min according to six-grade Riker sedation-agitation scale (RSAS)^14^ with peripheral oxygen saturation, SAP, DAP, OAB measurements. Postoperative pain severity was assessed by a visual analog scale (VAS; 0–10 cm) before departing the RSAS room. According to VAS (visual analog scale), if the patient's pain were 5 or more, an additional 0.5 mg/kg of petidine HCL (Aldolan®) IV was administered. Postoperative nausea and vomiting were recorded as “yes” or “no”. One day after the surgery, we also evaluated the patient wake-up satisfaction using the EVAN-G scale^15^ and the data about intraoperative awareness.

The primary aim of our study was to evaluate the patient satisfaction after surgery, while our second objective was to analyze intraoperative hemodynamic stability, intraoperative awareness occurrence, and postoperative pain and anxiety.

### Statistical analyses

In this study, the results of a descriptive analysis of the demographic data (age, weight, height, and BMI), gender and ASA classifications were used. The data was summarized using the mean and standard deviation. The Shapiro–Wilk test was used for the assumption of normal distribution of continuous variables. If variables were normally distributed, central tendency was expressed as the mean (SD). Means were compared using independent or paired Student's *t*-test. Spearman correlation analysis was used to find out a correlation between non-normally distributed independent variables. Fisher exact test was used for categorical data and expressed in count, percentages. Differences were considered significant if *p *< 0.05. Statistical analysis was performed using SPSS 22 (Chicago, IL, USA).

## Results

This study was carried out with 120 patients divided in two groups of 60 people. There was no statistically significant difference between groups in terms of demographic characteristics (age, BMI, gender, ASA) (*p *> 0.05) (Table 1). The operation periods of the groups were similar (*p* > 0.05) (Table 1).

**Table 1:** Comparison of group M and group C based on demographic parameters, hemodynamic parameters and surgical interventions characteristics.

Variable	Group M (*n* = 60)	Group C (*n* = 60)	*p*-Value^b^
Age, mean ± SD years^a^	31.93 ± 8.67	31.56 ± 8.05	0.81
Gender (male/female)	29/31	27/33	0.71
BMI, mean ± SD (kg/m^2^)^a^	25.47 ± 3.98	26.81 ± 4.76	0.98
ASA-PS class			0.14
Class I	23 (38.3%)	28 (46.6%)	
Class II	37 (61.6%)	32 (53.3%)	
Time of surgery, mean ± SD^a^	139.75 ± 9.75	141.03 ± 10.8	0.49
Time of anesthesia, mean ± SD^a^	159.58 ± 8.88	160.60 ± 9.17	0.53
MAP, mean ± SD (mm Hg)^a^	85.13 ± 10.43	86.66 ± 10.78	0.578
HR, mean ± SD (beat/min)^a^	78.56 ± 9.66	79.63 ± 13.84	0.730

ASA-PS, American Society of Anesthesiologist Physical Status; SD, standard deviation; Group M, music intervention; Group C, control group; MAP, mean arterial pressure; HR, heart rate.

a Values are expressed as mean (SD).

b Independent *t* test.

The most preferred music by our patients was Turkish pop music (29 cases). Eastern and Western music were selected by 20 and 9 patients, respectively. The anesthesiologist chose classical music for 2 patients who did not show a particular preference.

When the values of MAP, SAP and DAP were compared, the differences between the two groups were not statistically significant (*p *> 0.05), although the general results were lower in the music group.

When both groups were evaluated with RSAS, the results were lower in the music group (3.76 ± 1.64 vs. 5.11 ± 2.13), which means that the patients in the music group had a better awakening quality (*p *< 0.001) (Table 2).

**Table 2:** Effects of music therapy on recovery quality, VAS during recovery, patient satisfaction and intraoperative awareness.

Parameters	Group M (*n* = 60)	Group C (*n* = 60)	*p*^b^
*Ricker scale (quality of recovery)*^a^	3.76 ± 1.64	5.11 ± 2.13	<0.001
<5	44 (73.3%)	25 (41.6%)	
≥5	16 (26.6%)	35 (58.3%)	
*VAS during recovery*^a^	2.73 ± 1.28	3.61 ± 1.40	<0.001
<5	56 (93.3%)	51 (85%)	
≥5	4 (6.6%)	9 (15%)	
*Patient satisfaction*	44 (73.3%)	22 (36.6%)	<0.001
*Intraoperative awareness*	4 (6.6%)	9 (15%)	0.14

VAS, visual analog scale; Group M, music intervention; Group C, control group.

a Values are expressed as mean (SD).

b Independent *t* test.

The mean VAS score for pain was lower in the music group, showing statistical significance (2.73 ± 1.28 vs. 3.61 ± 1.40) (*p *< 0.001). Patients with severe postoperative pain (VAS ≥ 5) were medicated with 0.5 mg/kg Petidin HCL (Aldolan®) (4 patients of the music group vs. 9 of the control group) (Table 2).

Patient satisfaction rate was significantly higher in the music group (73.3% vs. 36.6%) than the control group (*p *< 0.001) (Table 2).

The incidence of intraoperative awareness was higher in the control group (4 cases vs. 9 cases), but the difference was not statistically significant (*p *= 0.14) (Table 2).

## Discussion

Music therapy, a nonpharmacological modality; can be accepted as an effective method intraoperatively and postoperatively when applied to septorhinoplasty patients under general anesthesia. In our study, we have concluded that, the patients in the music therapy group had better awakening quality (3.76 ± 1.64 vs. 5.11 ± 2.13; *p* < 0.001) and lower level of pain according to a VAS (visual analog scale) (2.73 ± 1.28 vs. 3.61 ± 1.40; *p* < 0.001), and higher patient satisfaction rates (73.3 vs. 36.6, *p* < 0.001). We have also deduced that the incidence of HR (heart rate), SAP (systolic arterial pressure), DAP (diastolic arterial pressure), MAP (mean arterial pressure) and intraoperative awareness were lower but not statistically significant.

Music therapy is one of the most effective therapeutic practices that draws attention of individuals from themselves and from their problems to another direction. Studies have shown that the relaxation quality of music is a non-invasive method that reduces the physiological effects of stress such as anxiety, blood pressure, heart rate, respiration rate and improves the emotional state of the patients.^13^, ^16^, ^17^

If two audible stimuli at different frequencies (1–30 Hz) are applied to both ears at the same time, they are perceived as single warning. This warning is described as a brainstem reaction originating from the superior olivary nucleus in both cerebral hemispheres, and this response is thought to lead to a hemispheric synchronization. It has been suggested that hemispheric synchronized sounds can be used for pain control, stress and anxiety treatment. Recorded CD's for this purpose are commercially marketed worldwide under the name of “nonpharmacologic surgical support”.^18^ In addition to music therapy, hemispheric synchronized sounds were also investigated for effects on BIS values in patients undergoing general anesthesia, but hemispheric syncing did not affect BIS values in patients receiving general anesthesia. More clinical trials are needed in this regard.^19^

Bondoc et al.^18^ investigated the effect of hemispheric sound on perioperative analgesic requirement in the preoperative and intraoperative periods of patients undergoing general anesthesia. In this study, patients were randomly divided into three groups: hemispheric synchronized audiophiles, their favorite music tracks or empty cassette listeners (control). Fentanyl was used as an analgesic during induction and intraoperative period. In the hemispheric voice group, there was less need for fentanyl than in the control or music group. Pain level and postoperative analgesic requirement were lower in the hemispheric group than in the other groups. It has also been found that the duration of stay until discharge from the hospital was shortened in the hemispheric voice group. However, there was no difference between the groups in terms of intraoperative heart rate, blood pressure levels and postoperative nausea, vomiting. In our study, we administered remifentanil infusion instead of fentanyl infusion during intraoperative analgesia and we concluded that remifentanil consumption was significantly reduced in the music group, similarly to the study of Bondoc et al.

Allen et al.^20^ reported that perioperative music therapy reduced the stress-induced hypertensive response in a group of geriatric patients to be undergoing ophthalmic surgeries under local anesthesia. The heart rates, systolic and diastolic blood pressures of the patients listening to the music were found to be similar to those measured one week before surgery. In this study, it was thought that the reason for the positive effect in the hemodynamic parameters was the reduction of the anxiety regarding the surgery, by redirecting the attention of the patient to the music. In addition, it has been observed that music increases the feeling of personal control in patients at postoperative conditions and that it leads to a general feeling of well-being. In our study, it was observed that intraoperative musical stimulation reduced the levels of HR, SAP, DAP, MAP but it was not statistically significant compared to the control group. Similarly to our study, there are other studies showing that music therapy has no effect on hemodynamic parameters.^18^, ^19^, ^21^

In an animal study carried out in order to explain the effect of music therapy on hemodynamics, the music showed to reduce blood pressure in hypertensive rats. Music-therapy applied to rats also showed that blood calcium levels increased, with consequent increase of dopamine synthesis in the brain via a calmodulin-dependent system. The increase in dopamine level is thought to decrease blood pressure by inhibiting sympathetic activity by D2 receptors.^22^ Based on these findings, it can be postulated that in diseases with dopaminergic dysfunction music therapy may be effective in relieving the symptoms. As a result of music therapy in Parkinson's disease, positive developments have been reported in motor and emotional functions and also in daily activities.^23^ Similarly, it has been observed that it relieved the symptoms of epilepsy.^22^

It has been reported that anxiolytic effects of music were investigated as a treatment modality in eliminating preoperative anxiety in anesthesiology practice.^11^, ^24^ Minimizing anxiety in the preoperative period facilitates the induction of anesthesia, prevents undesired reflex cardiovascular response, and reduces the required anesthetic dose by reducing oxygen consumption. In a study comparing preoperative music therapy with midazolam treatment, the reduction in anxiety score with music therapy was found to be significantly higher than with midazolam treatment.

In order to investigate the effect of music on anxiety in the preoperative period, 99 patients undergoing day surgery, were randomly divided into music and control groups. None of the patients were premedicated with a pharmacological agent for sedation. On the day of the surgery, any kind of music CD selected and brought by patients was listened to 30 min during the preoperative period. Because the study was double-blind, in the control group, the CD player played a blank CD. The anxiety levels of the patients were evaluated with the 40 item State/Trait Anxiety Inventory before and after this application. In addition; measurements of serum cortisol and catecholamine levels being neuroendocrine variables of anxiety, blood pressures and heart rate being physiological indicators of anxiety were also performed simultaneously. As a result, it was observed that music therapy reduced anxiety, but did not affect hemodynamic parameters such as blood pressure, heart rate, and serum cortisol and catecholamine levels.^10^ We did not evaluate the preoperative or postoperative anxiety in our study. We used the Riker sedation agitation scale (RSAS), a commonly used scale, to measure the level of postoperative sedation and concluded that the sedation scores of the patients in the music group were higher than those of the control group, similarly to other studies in the literature.

Koç et al.^13^ found that music therapy significantly reduces anxiety and BIS values and reported that, in addition to the need of less sedative drugs during regional anesthesia, listening to classical Turkish music could be a harmless, fun and low cost adjuvant. Music therapy has been shown to reduce the need for propofol to provide adequate sedation in patients with controlled sedation undergoing urological surgery (under spinal anesthesia). The author also observed that music therapy decreased 44% of the need for opioid consumption in patient controlled analgesia.^25^ It is clearly known that postoperative pain causes undesirable clinical conditions such as metabolic and endocrine responses, adverse effects on organ functions, muscle spasms and atelectasis. For this reason, postoperative analgesia management is highly vital. Nilsson et al.^12^ found that music therapy reduced the pain and analgesic drugs requirement. They studied 90 patients who underwent abdominal hysterectomy under general anesthesia randomly divided into three groups; music, therapeutic suggestion with music and control group. Relaxing music accompanied by wave sounds was listened to the music group. The same music was listened to the patients in therapeutic suggestion group in addition to a relaxing and encouraging suggestion. The suggestion was performed with a relaxing male voice telling that there would be no post-operative pain, nausea and vomiting, recovery would be quick, while patients in the control group listened to a pre-recorded tape containing noises of an active operation room. The follow-up showed that patients in the music group presented less pain and analgesic requirements, and recovered earlier than the others. In addition, fatigue sensation at discharge were less frequently observed in the groups of music and therapeutic suggestion with music. However; music therapy did not reduce postoperative nausea and vomiting. In this study, as in our study, patient-controlled analgesia was used as a routine technique in the treatment of pain, and similarly, the amount of analgesic consumed in the music group was found to be lower. In addition, postoperative nausea and vomiting in our study did not differ between the music and control groups. Also, we observed that in the music group, early postoperative parameters and sedation scores were positively affected.

In a study published in 1995 at CHEST, two groups were established to investigate the effect of music on patients undergone bronchoscopy. The music group had 21 patients the control group, 28 patients. It was concluded that the rate of satisfaction was higher in the music group.^26^ Another similar study consisted of patients submitted to colonoscopy under general anesthesia (85 patients in the music group vs. 81 patients in the control group). The rate of satisfaction in the music group was higher than in the control group (96.3% vs. 56.1%, respectively) *p* < 0.0001.^27^ In a meta-analysis published in 2019, on 8 randomized trials involving 712 patients under general anesthesia, satisfaction was significantly higher in the music group.^28^ In addition, other studies have confirmed that music has positive effects on patient satisfaction.^29^, ^30^

Finally, in our study, we examined the incidence of intraoperative awareness with music therapy. In a study that Mohamed Kahloul et al.,^31^ they examined 140 patients who underwent abdominal surgery under general anesthesia. Patients were divided in 2 groups of 70 patients, with and without music therapy. Patients without music therapy presented significative more episodes of intraoperative awareness. In our study, the incidence of intraoperative awareness was higher in the control group (4 patients in the music group vs. 9 patients in the control group) but not statistically significant (*p *> 0.05).

## Conclusion

We have found that music therapy decreases the pain level and the need of analgesic drugs intake intraoperatively and postoperatively. In addition, we showed that it has positive effects on postoperative parameters and level of sedation. In conclusion, we have shown that music therapy is a non-pharmacological method with practically no costs, easy-to-apply, without side effects, that increases the sedation and reduces pain levels, as observed in previous clinical trials. However, the experience on this subject is still very limited despite the increasing number of trials. More efforts are needed to ensure that music therapy gains a more respectable and distinct place in the modern health care system. There is also a need for prospective clinical trials involving more patients, multicentered, double-blinded, randomized and controlled, with blood tests investigation. We also think that human and animal studies would be useful to define the different mechanisms of action that would explain the positive effects of music therapy.

## Funding

The study was funded by departmental resources.

## Conflicts of interest

The authors declare no conflicts of interest.

## References

[1] Flanagan D.A., Kerin A. (2017). How is intraoperative music therapy beneficial to adult patients undergoing general anesthesia? A systematic review. Anesthesia J.

[2] Nilsson U. (2008). The anxiety and pain-reducing effects of music interventions: a systematic review. AORN J.

[3] Shafer A., Fish M.P., Gregg K.M., Seavello J., Kosek P. (1996). Preoperative anxiety and fear: a comparison of assessments by patients and anesthesia and surgery residents. Anesth Analg.

[4] Biebuyck J.F.W.C. (1990). The metabolic response to stress: an overview and update. Anesthesiology.

[5] Desborough J.P. (2000). The stress response to trauma and surgery. Br J Anaesth.

[6] Kayhan Z. (2004). Clinic anesthesia 3. Edition Istanbul. Logos Publ.

[7] Allred K.D., Byers J.F., Sole M.L. (2010). The effect of music on postoperative pain and anxiety. Pain Manag Nurs.

[8] Buffum M.D., Sasso C., Sands L.P., Lanier E., Yellen M.H.A. (2006). A music intervention to reduce anxiety before vascular angiography procedures. J Vasc Nurs.

[9] Fischer S.P., Bader A.M.S.B., Miller R.D. (2008). Miller‘s anaesthesia.

[10] Wang S., Kulkarni L., Dolev J., Kain Z. (2002). Music and preoperative anxiety: a randomized, controlled study. Anesth Analg.

[11] Bringman H., Giesecke K., Thörne A., Bringman S. (2009). Relaxing music as pre-medication before surgery: a randomised controlled trial. Acta Anaesthesiol Scand.

[12] Nilsson U., Rawal N., Uneståhl L.E., Zetterberg C., Unosson M. (2001). Improved recovery after music and therapeutic suggestions during general anaesthesia: a double-blind randomised controlled trial. Acta Anaesthesiol Scand.

[13] Koç H., Erk G., Apaydın Y., Horasanlı E., YiğitbaĢı B.D.B. (2009). Epidural anestezi ile herni operasyonu uygulanan hastalarda klasik türk müziğinin intraoperatif sedasyon üzerine etkileri. Türk Anest Rean Der Derg.

[14] Muir W.W., Robertson J.T., Kb B.A., Ferragut R., Unidad C.M., Intensivos C. (2012). Prospective evaluation of the Sedation-Agitation Scale for adult critically ill patients. Colomb J Anesthesiol.

[15] Auquier P., Pernoud N., Bruder N., Simeoni M.-C., Auffray J.-P., Colavolpe C. (2005). Development and validation of a perioperative. Anesthesiology.

[16] Migneault B., Girard F., Albert C., Chouinard P., Boudreault D., Provencher D. (2004). The effect of music on the neurohormonal stress response to surgery under general anesthesia. Anesth Analg.

[17] Cook J.D. (1981). The therapeutic use of music: a literature review. Nurs Forum.

[18] Dabu-Bondoc S., Nadivelu N., Benson J., Perret D.K.Z. (2010). Hemispheric synchronized sounds and perioperative analgesic requirements. Anesth Analg.

[19] Dabu-Bondoc S., Drummond-Lewis J., Gaal D., McGinn M., Caldwell-Andrews A.A.K.Z. (2003). Hemispheric synchronized sounds and intraoperative anesthetic requirements. Anesth Analg.

[20] Allen K., Golden L.H., Izzo J.L., Ching M.I., Forrest A., Niles C.R. (2001). Normalization of hypertensive responses during ambulatory surgical stress by perioperative music. Psycosom Med.

[21] Nilsson U., Rawal N., Enqvist B., Unosson M. (2003). Analgesia following music and therapeutic suggestions in the PACU in ambulatory surgery: a randomized controlled trial. Acta Anaesthesiol Scand.

[22] Sutoo D., Akiyama K. (2004). Music improves dopaminergic neurotransmission: demonstration based on the effect of music on blood pressure regulation. Brain Res.

[23] Pacchetti C., Mangini F., Aglieri R., Fundaro C.M.E.N.G. (2000). Active music therapy in Parkinson‘s disease: an integrative method for motor and emotional rehabilitation. Psychosom Med.

[24] Twiss E., Seaver J., McCaffrey R. (2006). The effect of music listening on older adults undergoing cardiovascular surgery. Nurs Crit Care.

[25] Koch M.E., Kain Z.N., Ayoub C.R.S. (1998). The sedative and analgesic sparing effect of music. Anesthesiology.

[26] Dubois J.M., Bartter T., Pratter M.R. (1995). Music improves patient comfort level during outpatient bronchoscopy. Chest.

[27] Bechtold M.L., Perez R.A., Puli S.R., Marshall J.B. (2006). Effect of music on patients undergoing outpatient colonoscopy. World J Gastroenterol.

[28] Bechtold M.L., Puli S.R., Othman M.O., Bartalos C.R., Marshall J.B., Roy P.K. (2009). Effect of music on patients undergoing colonoscopy: a meta-analysis of randomized controlled trials. Dig Dis Sci.

[29] Bradley Palmer J., Lane D., Mayo D., Schluchter M., Leeming R. (2015). Effects of music therapy on anesthesia requirements and anxiety in women undergoing ambulatory breast surgery for cancer diagnosis and treatment: a randomized controlled trial. J Clin Oncol.

[30] Jayaraman L., Sharma S., Sethi N., Jayashree S., Kumra V.P. (2006). Does intraoperative music therapy or positive therapeutic suggestions during general anesthesia affect the postoperative outcome? A double blind randomised controlled trial. Indian J Anaesth.

[31] Kahloul M., Mhamdi S., Nakhli M.S., Sfeyhi A.N., Azzaza M., Chaouch A. (2017). Effects of music therapy under general anesthesia in patients undergoing abdominal surgery. Libyan J Med.

